# Mathematical modeling for developmental processes

**DOI:** 10.1111/dgd.12856

**Published:** 2023-05-26

**Authors:** Yoh Iwasa

**Affiliations:** ^1^ Department of Biology, Faculty of Science Kyushu University Fukuoka Japan; ^2^ Institute for Freshwater Biology Nagano University Ueda Nagano Japan

**Keywords:** deformation tensor, feedback vertex set, forces, gene regulatory network, optimal control

## Abstract

We review several mathematical models and concepts in developmental biology that have been established over the last decade. (1) Feedback vertex set: Ascidian embryos contain cells of seven types, and cell fate is controlled by ~100 interacting genes. The “feedback vertex set” of the directed graph of the gene regulatory network consists of a small number of genes. By experimentally manipulating them, we can differentiate cells into any cell type. (2) Tissue deformation: Describing morphological changes in tissues and relating them to gene expression and other cellular processes is key in understanding morphogenesis. Expansion and anisotropy of the tissue are described by a “deformation tensor” at each location. A study on chick limb bud formation revealed that both the volume growth rate and anisotropy in deformation differed significantly between locations and stages. (3) Mechanobiology: Forces operating on each cell may alter cell shape and gene expression, which may subsequently exert forces on their surroundings. Measurements of force, tissue shape, and gene expression help us understand autonomous tissue deformation. (4) Adaptive design of development: An optimal growth schedule in fluctuating environments explains the growth response to starvation in *Drosophila* larvae. Adaptive placement of morphogen sources makes development robust to noises.

## Graphical abstract

In this paper, after quickly mentioning classical models of morphogenesis, we explain key aspects of the following four mathematical tools that help us understand developmental processes: (1) gene regulatory networks of cell differentiation in tunicate embryos, (2) the deformation tensor in chick limb bud formation, (3) mechanobiology, and (4) adaptive design in *Drosophila* larval growth/pupation. These concepts have been formulated in Japan in the last 10–15 years.

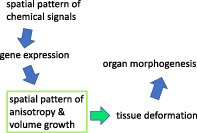

## 1 INTRODUCTION

Many diverse mathematical models have been studied to analyze morphogenesis, pattern formation, and various developmental processes (Morelli et al., 2012). For example, one group of mathematical models considers the morphology formed by the repeated addition of a unit structure. The Raup model for molluscan shells generates coiled patterns corresponding to diverse shapes of shells with only a few parameters (Raup, 1962, 1966; Raup & Michelson, 1965), which can be considered an outcome of adding a ring‐like structure that is slightly larger and slightly twisted compared to the previous one (Hammer & Bucher, 2005; Noshita, 2014; Noshita et al., 2016). The Lindenmayer system (L‐system) generates multicellular structures by repeatedly replacing a cell with one or more cells, possibly including branching (Lindenmayer, 1968a, 1968b; Prusinkiewicz & Lindenmayer, 1990). In the 1970s, Hisao Honda published a series of papers on the shape of trees (Honda, 1971; Honda & Fisher, 1978), in which one branch was spliced by another of reduced length at some angle to the last one. This model can be used to generate diverse tree shapes.

Another simple concept is cell sorting (Mochizuki et al., 1996, 1998; Steinberg, 1963). If cells of two different types are placed randomly on a flat plane, they engage in random movement and exchange locations with their neighbors. If the cell‐to‐cell adhesion force is stronger within the same cell type than between different cell types, the cells spontaneously form clusters of the same cell type. A similar process was used to explain the formation of tiling patterns in the fish retina. Four photoreceptor cells, or cone cells, form a regular pattern called the cone mosaic, which differs between zebrafish and medaka. A random assortment can generate a cone mosaic if the cell‐type‐dependent adhesion strength is chosen appropriately (Mochizuki, 2002).

The most influential mathematical concept in developmental biology is the Turing model (Turing, 1952). It describes the formation of heterogeneous spatial patterns, such as spot patterns or labyrinth patterns, in a perfectly homogeneous medium if the cell‐to‐cell interaction is activating between cells in the short range but inhibiting in the middle range (see also Gierer & Meinhardt, 1972; Meinhardt, 1982). The model has inspired numerous mathematical studies (Kondo & Miura, 2010; Murray, 1989; Raspopovic et al., 2014). Shigeru Kondo and colleagues demonstrated that the skin patterns of marine angelfish change with body size and in response to experimental manipulation (Kondo & Asai, 1995; Nakamasu et al., 2009). After experimental attempts to identify the mechanistic basis of the formation of zebrafish skin patterns, they concluded that the mechanism responsible for the diverse skin patterns was direct cell‐to‐cell contact, instead of dispersing chemical signals as previously assumed (Nakamasu et al., 2009; Watanabe & Kondo, 2015; Yamanaka & Kondo, 2014).

A wide variety of simulators for generating tissue and organ shapes have been investigated. An example is the cellular Potts model (Graner & Glazier, 1992; Glazier & Graner, 1993; Scianna & Preziosi, 2013: Hirashima et al., 2009, 2017). Cell‐center dynamics assume that cells are subjected to a force, which is a function of the distance between neighboring cells (Merks & Glazier, 2005; Morishita & Iwasa, 2008a). Cells may proliferate or interact with other cells by exchanging chemical signals and exerting mechanical forces. In vertex dynamics, the basic agents that interact with each other and form patterns are the boundaries between cells (Farhadifar et al., 2007; Fletcher et al., 2014; Honda & Nagai, 2022). The vertex dynamics model was derived from the previous boundary‐shortening model for cell‐sheet patterns (Honda & Eguchi, 1980), and can handle cell intercalation and convergent extension (Honda & Nagai, 2022).

Several other methods exist for describing and analyzing morphological changes during development. We will not explain them here, because they were well established many years ago and can be found in excellent textbooks. In the following sections, we will focus on four mathematical models that have been developed in the last 10–15 years by researchers who began their academic careers in the mathematical biology group at Kyushu University.

## 2 FEEDBACK VERTEX SET IN GENE REGULATORY NETWORKS

Cell differentiation is an important process in the development of multicellular organisms. Specific types of cells, such as epithelial, nerve, endoderm, and muscle cells, are induced from undifferentiated cells. Many genes are involved in determining cell fate. Atsushi Mochizuki and colleagues have developed a new theory that captures the key aspects of the dynamics of gene regulatory networks. Subsequently, this theory was tested experimentally using a gene regulatory network for cell fate specification in ascidians, or tunicates.

**FIGURE 1:**
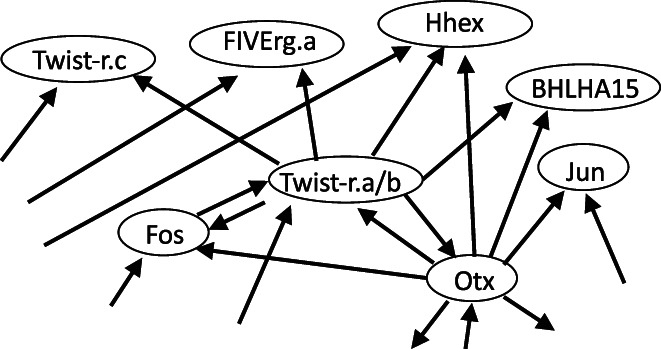
Illustration of the regulatory network of genes determining the differentiation of cells in tunicate embryos. This shows a small portion of the network in Figure 2a in Kobayashi et al. (2018). The original network includes 92 nodes and 328 arrows. Nodes indicate factors (genes) and arrows indicate the interactions between them.

Tunicates are marine animals with a short planktonic larval stage and sessile adults. A tunicate larva has a simple body structure and contains tissues of seven types, including epidermis, brain, muscle, and endoderm. Figure 1 shows a small portion of the gene regulatory network that determines cell fate (Kobayashi et al., 2018). The entire network is comprised of 92 factors (genes) and 328 interactions between them. This was a summary of the study by Noriyuki Satoh and colleagues (Imai et al., 2006; Satou et al., 2009).

Gene regulatory networks are represented as directed graphs. Nodes are genes and are connected by arrows (or directed edges). The graph is constructed from the information on gene regulation as follows: we assume that gene i is affected by a different gene j. The rate of change in the expression of gene i depends on the expression of gene j. Therefore, the differential equation for the expression level of gene i depends on the expression level of gene j. Then, we draw an arrow from j to i. We do not distinguish between inhibition and activation. Similarly, neither the strength of the regulation nor its functional form is important. The arrow simply indicates that the expression level of gene j can affect gene i.

We consider a set of differential equations (i.e., dynamics) for gene expression levels in the regulatory network. Many cells in an ascidian embryo share the same genome. This implies that the dynamics of gene expression in different cells follow the same set of differential equations. During development, each cell differentiates into one of the following seven cell types: epidermis, brain, neural tissue, endoderm, notochord, mesenchyme, and muscle. Different cell types correspond to different stable equilibria of the dynamics of gene expression levels. Hence, we expect that the dynamics have at least seven stable equilibria.

The first question we ask is regarding the amount of information to determine whether a cell will differentiate into a particular cell type. Do we have to measure the expression levels of all the genes included in the network, which contains 92 genes based on experimental data? Instead, there might be a small number of key genes, and by simply measuring their expression levels, we can accurately identify the developmental fate of the cell.

The second question is regarding the way to manipulate the expression of genes to control a given cell to differentiate into a particular cell type which we choose. Do we have to manipulate all 92 genes in the regulatory network? By contrast, there might be a small number of key genes, and we can make a given cell differentiate into a particular cell type by manipulating these key genes.

**FIGURE 2:**
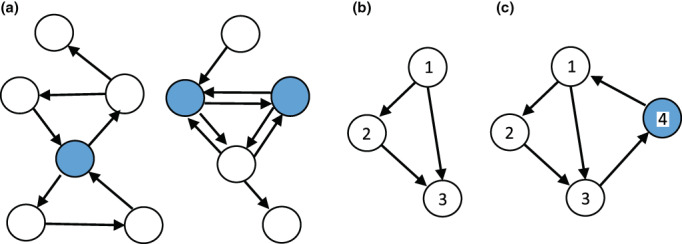
Examples of directed graphs indicating simple gene regulatory networks. (a) Feedback vertex sets in two simple directed graphs. Colored nodes indicate the feedback vertex sets. If we remove them, the remaining graph has no cycle. (b) A simple directed graph without cycles. (c) A simple directed graph with cycle. There are two cycles: 1234 and 134. Node 4 is an FVS, because removal of this node results in a graph without cycles.

A relatively small number of genes may provide the answer to both of these questions. They are called “feedback vertex set” genes (abbreviated as FVS genes) (Fiedler et al., 2013; Mochizuki et al., 2013). An FVS is defined as a set of nodes in a directed graph whose removal results in a graph without directed cycles. Figure 2a shows two simple directed graphs, which have cycles. If the blue nodes are removed, the remaining directed graphs have no cycles. Hence, the blue nodes form an example of an FVS. There can be multiple choices of FVS. Suppose that we are given a gene regulatory network for determining cell fate and that the network contains all the genes and their interactions affecting cell differentiation (i.e., the network is perfect). We then identify an FVS by following a standard procedure in graph theory. Mathematics tells us that by manipulating the genes in the FVS, we should be able to generate all possible cell states.

### 2.1 Experimental tests with ascidian larva

Before explaining the reasoning underpinning these methods in an intuitive manner in the next section, we will demonstrate that they are biologically useful. Yutaka Satou and colleagues have performed an experimental test of this theory using larvae of tunicates, or ascidians. From the gene regulatory network shown in Figure 1 (Kobayashi et al., 2018), they first removed nodes without any inputs and nodes without any outputs because they are not involved in any cycle. To the obtained simplified network, they applied the same node‐removing procedure. After repeatedly applying the operation, finally they found minimum FVSs containing only five genes. When multiple choices exist for the FVS, the one with the smallest number of nodes is the most useful.

The top row of Table 1 illustrates one choice of five FVS factors (Foxa.a, Foxd, Neurogenin, Zic‐r, and ERK signaling). Satou and colleagues experimentally manipulated the activity of these five factors by either activating or inhibiting them. The embryos were then cultured, and the expression of marker genes corresponding to the seven tissues was measured. If all five factors were downregulated in the FVS while other factors remained uncontrolled, only the epidermal marker was observed in all the manipulated embryos. If they manipulated the genes in a different manner, for example, they upregulated Zic‐r and ERK signaling and downregulated Foxa.a, Foxd, and Neurogenin, without affecting other genes, all the cells in the manipulated embryos showed expression of the mesenchymal marker. They performed these experiments for $2^{5}=32$ combinations. They could produce six out of seven cell types (Kobayashi et al., 2018). This result is extremely impressive, as they produced most cell types.

**TABLE 1:** A small portion of the results of Kobayashi et al. (2018).

Foxa.a	Foxd	Neurogenin	Zic‐r.b	ERK signaling	Cell type
Down	Down	Down	Down	Down	Epidermis
Up	Down	Down	Down	Down	Endoderm
Down	Up	Down	Down	Down	Notochord
Down	Down	Up	Down	Up	Neural tissues
Down	Down	Up	Up	Down	Brain
Down	Down	Down	Up	Up	Mesenchyme
Up	Down	Up	Up	Down	Neural tissues
Up	Up	Up	Down	Up	Notochord

*Note*: The top row indicates five genes in the feedback vertex set. Rows indicate the experimental manipulation of these five factors (genes) as a combination of up‐/downregulation, and the rightmost column indicates the cell types into which the manipulated cells differentiated. The entire table includes $2^{5}=32$ rows. Kobayashi et al. generated cells of six of the seven cell types in tunicate embryos. However, they failed to produce muscle cells.

However, they could not produce muscle cells, which is inconsistent with the theory that all seven cell types should be produced if FVS genes are manipulated appropriately. In a subsequent study, Kobayashi et al. (2021) explored the possibility that the gene regulatory network might have included missing regulations between genes. Because there were close to 100 genes and many interactions involved in the network, re‐examining the system exhaustively would be extremely difficult. Adding a single arrow to the regulatory network may change the FVS. These were considered good candidates for unknown regulation. They then studied these candidates experimentally and discovered one between‐gene interaction that was not included in the original network (Kobayashi et al., 2021). The revised network contained six FVS genes and with this network all seven cell types could be produced experimentally. This is a great achievement: it demonstrated that mathematical theory can help identify missing interactions between genes.

### 2.2 Logic underpinning the method

In this section, we would like to explain the reasoning underpinning this method. The most important proposition is that the dynamics of gene expression levels converge to a single stable equilibrium if the corresponding network (directed graph) contains no cycles. For this hypothesis to hold, we must pay attention to the way in which we draw the graph. In the following section, we explain this in a simplified manner (for exact conditions, please read the original papers: Mochizuki et al., 2013; Fiedler et al., 2013).

We consider two genes, i and j, whose expression levels are $x_{i}$ and $x_{j}$, respectively. If gene i is affected by the expression of gene j, we place an arrow from j to i. If the dynamics of gene expression are expressed by a set of differential equations ($dx_{i}/\mathrm{dt}=f_{i}\left(x_{1},\ldots ,x_{j}\right);i=1,2,\ldots ,n$), the arrow from j to i implies that $dx_{i}/\mathrm{dt}$ depends on $x_{j}$. This effect can be activation or inhibition. The arrow indicates a direct effect. Even if there is an indirect effect, for example, gene j affects k and k affects i, we do not place an arrow from j to i.

Another important assumption is that it requires an inequality called “decay condition,” which indicates that a partial derivative is negative ($\frac{\partial }{\partial x_{i}}\left(dx_{i}/\mathrm{dt}\right)<0$), implying that the rate of increase in gene expression becomes slower as the current expression level increases. This assumption is plausible for gene expression dynamics in many systems we encounter in developmental biology. If the decay condition holds for the dynamics of $x_{i}\left(t\right)$ (the expression level of gene i) and the dynamics of other genes are exactly the same, $x_{i}\left(t\right)$ will converge to a single stationary level, irrespective of its initial level. In exceptional cases, there might be a gene whose expression increases more rapidly when its current expression level is higher, indicating positive feedback. More likely is the situation in which the partial derivative shown above is negative, but its magnitude is very small; that is, the decay is extremely slow. In both cases, $x_{i}\left(t\right)$ does not converge to the same level, starting from different initial levels during the time appropriate for developmental phenomena, even if the dynamics of the other genes are exactly the same. We then add a self‐loop indicating that gene i affects itself.

We now consider one gene that is not affected directly by other genes in the same network. In addition, the decay condition holds (it has no self‐loop). Then we can represent it as an isolated node. The expression level of this gene should converge to a single final equilibrium. There is no possibility of multiple equilibria.

The same conclusion holds for a network of genes containing no cycles. Figure 2b illustrates an example of a network without cycle. Consider gene 1 with expression level $x_{1}$. Because of the decay condition, $x_{1}$ will converge to its final value ${\bar{x}}_{1}$. After $x_{1}$ converges to ${\bar{x}}_{1}$, $x_{2}$ also converges to its final value ${\bar{x}}_{2}$ because of the decay condition. After $x_{1}$ and $x_{2}$ converge to their respective final values, $x_{3}$ also converges to ${\bar{x}}_{3}$ because of the decay condition. Therefore, the entire system has a single final state, and the system converges to it, starting from any initial condition.

However, if a network contains a cycle of nodes, its dynamics can exhibit multiple stable equilibria. Figure 2c illustrates a directed graph, which contains a cycle 1234. Blue node 4 indicates an FVS gene, because if we remove node 4, the remaining graph 123 has no cycle.

Suppose that A and B are two different equilibria of the dynamical system corresponding to 1234 and that the expression level of gene 4 is high at A and low at B. Then, we experimentally manipulate gene 4 to be expressed at a high level equal to the stationary value of gene 4 at A. Then the expression levels of the three other genes converge to a single stable equilibrium, which must be equal to the values at equilibrium A. Alternatively, if we manipulate gene 4 to be expressed at a low level equal to that at equilibrium B, the other three genes converge to a single stable equilibrium, which must be the same as that at equilibrium B. Therefore, by experimentally manipulating the expression of gene 4 to be high or low, we can push the entire system into equilibrium A or B without controlling other genes.

We can apply the same rationale to networks that include more genes. Suppose that the gene regulatory network contains 92 nodes and one choice of FVS includes only five nodes. The network from which the FVS nodes were removed (containing 87 nodes) would have no cycle. Hence, every time the expression levels of the five FVS genes are fixed, the dynamics should converge to a single final state (equilibrium), irrespective of the initial condition of the other genes. This suggests that by appropriately manipulating the five FVS genes, we should be able to produce all the possible equilibria. This is the reasoning we adopted in the biological rationale described in the previous section.

We would like to emphasize that this argument concerns general nonlinear differential equations, although the conclusion depends only on the presence or absence of arrows or interactions between genes. This method is independent of the functional form of the interaction, the strength of dependence, or the direction of the effect. Simple algebraic properties of a system can lead to biologically useful predictions.

## 3 TISSUE DEFORMATION

**FIGURE 3:**
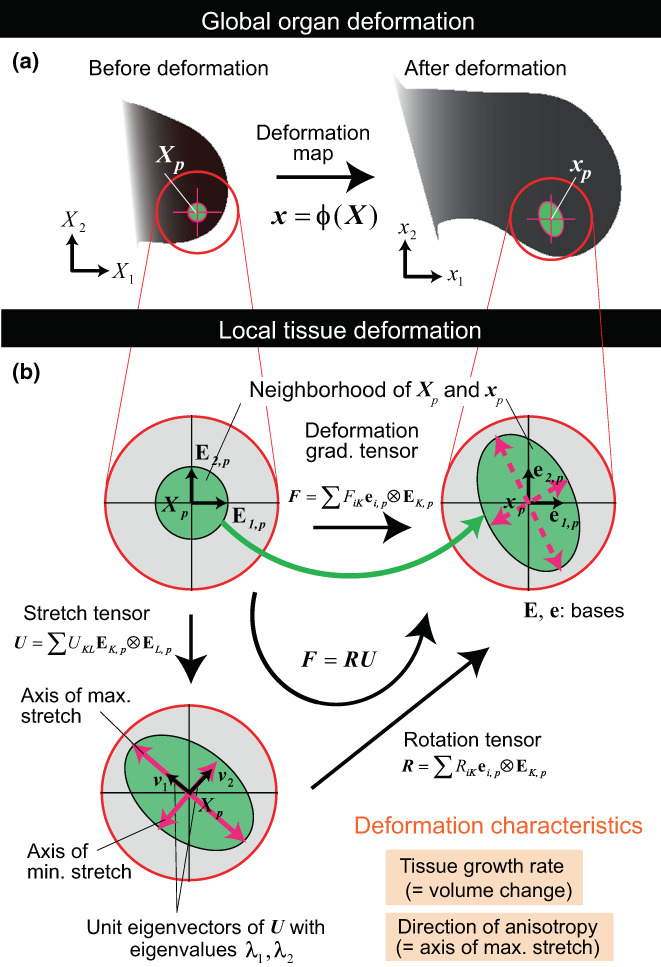
Tissue deformation. When an area element is deformed, the volume expansion and anisotropy (including the direction and magnitude of anisotropy) are described in the deformation gradient tensor measured at each location of the tissue. From this, the deformation tensor is calculated. From Figure 1 in Morishita and Suzuki (2014).

Describing the morphological changes in tissues and relating them to gene expression and other cellular processes is a key step toward understanding morphogenesis. The expansion and deformation of tissue can be described in terms of the “deformation tensor” at each location of the tissue (Figure 3). Yoshihiro Morishita and colleagues began with the following question: What is the simplest model explaining the protrusion of chick limb buds? This process is controlled by two diffusive chemical signals: fibroblast growth factor (FGF) and Sonic hedgehog (SHH), which have sources at two different positions. Each produces a concentration gradient of the chemical signal over the limb buds. Consequently, cells in the tissue receive these chemical signals at different concentrations. From these, cells receive information on their position in the tissue and they may be instructed on what to do next (move, differentiate, divide, or remain as is). Cells in a restricted area may start proliferating rapidly and cause localized volume growth (or volumetric growth), which may act as a force to protrude the limb bud. We could reproduce the morphogenesis and deformation of the tissue that appeared similar to the observed ones by specifying the localized position of high volume growth (Morishita & Iwasa, 2008a). Unfortunately, this idea turned out to be too simplistic, as explained below.

Figure 3 shows the morphological changes in areas resembling chicken limb development from stages 22 to 24 (n.b., actual chicken limb buds are not flat and have some thickness). A small area element expanded and the size of the area increased, indicating volume growth. We note that the magnitude of expansion in one direction is greater than that in the perpendicular direction. This is a phenomenon called “anisotropy,” indicating that the expansion rate is not equal in all directions. The volumetric growth and the magnitude and direction of anisotropy are represented in the “deformation tensor” evaluated at each location of the tissue (Morishita & Suzuki, 2014).

**FIGURE 4:**
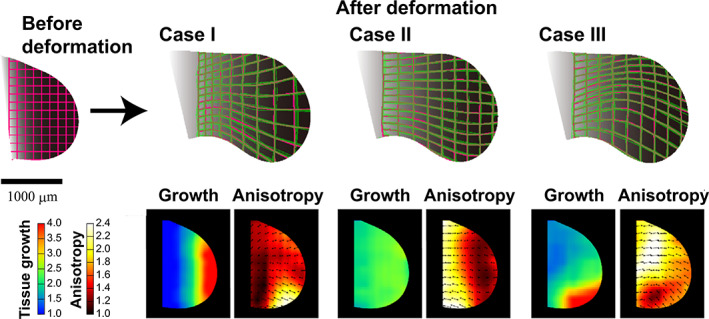
Spatial patterns of volume growth rate and anisotropy in tissue. Different combinations of volume growth rate and anisotropy can produce the same change in shape. From Figure 2a and b in Morishita and Suzuki (2014). Among these three cases, Case III most closely resembles the observed spatial distribution of high volume growth rates.

The same tissue deformation can be realized by different spatial distributions of the volume growth rate and anisotropy. Figure 4 illustrates the shape of the tissue imitating the limb bud before and after deformation. In Case I, the area of high volume growth rate is concentrated at the distal end. Case II shows a very similar transition of limb bud shape to Case I. However, in this case, volume growth occurs uniformly throughout the entire tissue. During this transition, the location of the high anisotropy and its direction at different locations in the tissue differ from those in Case I. In the third example, shown in Case III, the spatial distribution of the volume growth rate is still different from that in the other two cases. However, it produced morphological changes of the entire tissue similar to Cases I and II. Hence, even if we can explain morphogenesis by assuming a particular spatial distribution of high volume growth rate, this does not justify the assumed spatial distribution of volume growth. We must measure the spatial distributions of both the anisotropy and the volume growth rate (i.e., deformation).

Morishita and Suzuki (2014) developed a new method to measure the deformation tensor by using randomly placed sparsely injected markers and comparing two photographs at subsequent times. Using this method, they obtained reliable estimates of the deformation tensors at different tissue locations. Their method was robust as long as the deformation tensor changed over space slowly and smoothly.

During the deformation from stages 23 to 24 of chick limb bud formation, the area with high volume growth rate strongly overlapped with the area of high SHH gene expression. This suggests that high SHH expression is correlated with cellular processes that lead to rapid proliferation and volume growth (Morishita et al., 2015).

In a subsequent study, they reported that in stage 23, a high volume growth rate was observed in the posterior portion of the limb bud. However, in the earlier stages, a high volume growth rate was concentrated in the distal portion, whereas in the later stages, the volume growth rate was highest in a location very different from either of them (Morishita et al., 2015).

**FIGURE 5:**
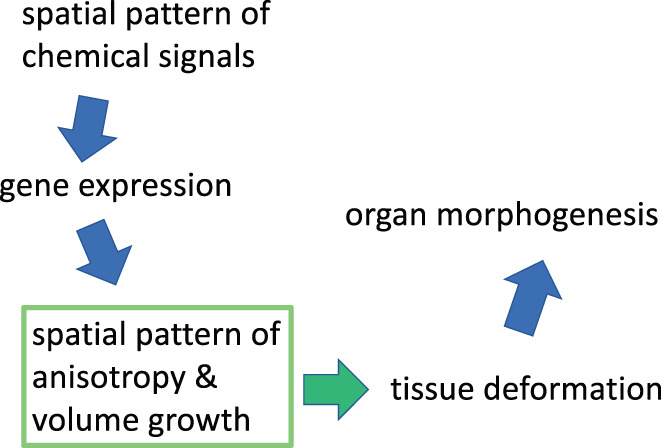
Scheme of the morphogenesis of tissues or organs. Measuring the deformation tensor plays an important role in understanding the changes in the shape of tissues or organs.

In summary, the local growth rate is not uniform but it may be very high in certain locations. The location of the high volume growth rate changes during development. Similarly, the local anisotropy also shows a spatiotemporal pattern over the tissue. They are combined into the deformation tensor at each location (Figure 5). Deformation is performed by cellular processes such as proliferation (frequency/division orientation), size/shape change, and rearrangement of cells. These are instructed by chemical signals. The spatial patterns of the deformation tensor (local anisotropy and local growth rate) can be measured from observed changes in tissue. On the other hand, the spatial pattern of the deformation tensor determines the way the tissue shape changes. Certainly, biological studies of each step in this chain of events must be performed.

The method explained above assumes that morphological changes occur on a flat plane. However, in some cases, the flat plane is not maintained, as in chick forebrain morphogenesis, which involves the deformation of epithelial sheets. The method has been expanded to the morphogenesis of the forebrain (Morishita et al., 2017) and heart (Kawahira et al., 2020).

## 4 MECHANOBIOLOGY

Cells receive input signals from other cells not only as signaling molecules but also as mechanical forces (Lenne et al., 2021; Ohtsuka et al., 2022). For example, Tsuyoshi Hirashima and colleagues examined the way in which epithelial cells mechanically interact with neighbors within a flat monolayer sheet and found that epithelial cells pull each other. The mechanical force functions as an input signal to the cell, which subsequently exhibits stretch‐induced activation of ERK signaling. The cell starts to contract and pull back its neighbors. Mechanical interactions between cells cause traveling waves of ERK activation and collective cell migration (Boocock et al., 2021; Hino et al., 2020). The spatiotemporal patterns of the traveling waves observed in the experiments were well explained by the predictions of a simple mathematical model.

Size control in biological tissues involves multicellular communication via mechanical forces during development, as demonstrated by studies on maintaining the diameter of an epithelial tube during murine epididymal development (Hirashima, 2022; Hirashima & Adachi, 2019). For the morphogenesis of the chick forebrain, see Ohtsuka et al. (2022).

These studies show that to understand the morphogenesis observed during development, we need to measure not only the gene expression level, but also the spatial pattern of chemical signals, the magnitude of deformation and forces operating between cells, and their changes over time. The morphogenesis of organs and tissues occurs through interactions between different processes. Mathematical models can combine this diverse information to provide a basic understanding of morphogenesis.

Mechanobiology is one of the most‐studied fields in recent years. It encompasses a variety of topics, from the cytoskeleton and force‐sensing receptors/channels to gene expression leading to force sensing/generation (Wen et al., 2021) and tissue morphogenesis (Okuda et al., 2018; Savin et al., 2011).

## 5 ADAPTIVE DESIGN OF DEVELOPMENT

In the last section, we would like to discuss the concept of adaptive design in developmental biology with two examples.

### 5.1 Optimal growth schedule

Basic ideas and analyses of the optimal growth schedules for multicellular organisms have been established in the field of ecology. Consider an annual plant, such as a cosmos. Seeds germinate at the beginning of the growing season. Starting from a small body size, the plant obtains material through photosynthesis and produces more leaves and roots, thereby increasing body size. Toward the end of the growing season, the plant discards them. It releases seeds that contribute to the subsequent generations. The phenotype that achieves the largest reproductive success through flowers and fruits is the one that wins in the competition with other phenotypes. After many generations, we should have plants that achieve their highest lifetime reproductive success, or fitness. This rationale justifies the use of an optimization model.

We may apply this rationale to the question of when an annual plant should start flowering and stop producing new leaves to achieve maximum fitness. Let us consider a plant composed of two parts: a production part and a reproductive part. The production part consists of leaves and roots, and a larger production part enhances the rate of photosynthesis. The reproductive part includes flowers and fruits, which do not contribute to photosynthesis. During the growing season, plants allocate materials obtained by photosynthesis between the two parts. We denote the fraction of reproductive allocation by $u\left(t\right)$ ($0\leq u\left(t\right)\leq 1$). This ratio may depend on t, the time within the growing season (0<t<T). Our question is what is the optimal schedule of allocation that attains the maximum total reproductive investment for the season.

Mathematically, this is an optimal control problem (Cohen, 1971; Iwasa, 2000). We can solve the problem by Pontryagin's maximum principle or dynamic programming. The answer is simple: there is a switching date. Before switching, the plant should invest all its resources in leaves and roots, rather than in reproduction. After switching, no new leaves are produced and all income should be allocated to flowers and fruits ($u\left(t\right)=0$ for $t<t_{s};$ $u\left(t\right)=1$ for $t>t_{s}$). Many physiological processes of a plant change when transitioning from vegetative to reproductive growth. The date of switching is controlled by the key plant hormone, FLOWERING LOCUS T (Wigge et al., 2005).

Importantly, the optimal date of flowering depends critically on the productivity of the environment. Under good light conditions with abundant soil moisture, plants enjoys fast photosynthetic rates. The plant then starts flowering late: it continues to produce leaves until the end of the growing season and then produces many seeds. By contrast, in dark environments, the plant stops leaf production and begins flowering earlier. This prediction of the simple optimization model is close to the observed growth behavior of annual plants.

**FIGURE 6:**
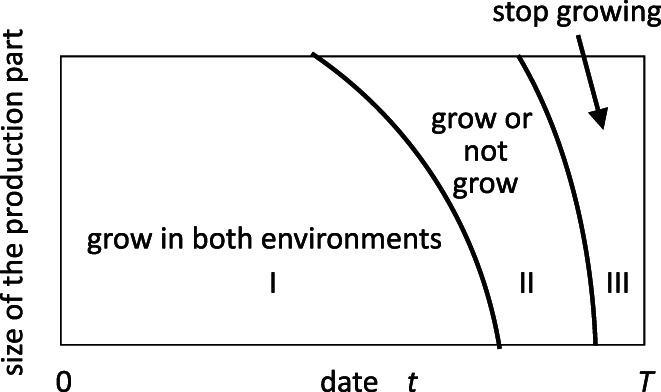
Optimal growth schedule of an annual plant in a fluctuating environment. The vertical and horizontal axes indicate plant size and the date in the growing season, respectively. The length of the growing season is T. The growth rate changes between high and low values at random times. The plant starts growing from a small initial size. In the region labeled as I, the plant should produce leaves and roots without investing in reproductive activity in both environments. In region II, the plant should grow if the current environment is good, whereas it should stop growing and start accumulating resources if the current environment is bad. In region III, the plant should not invest in leaves or roots in either environment (modified from Iwasa, 1991).

We may consider an optimal schedule if the productivity (e.g., the light availability) of the environment switches between high and low in an unpredictable manner (Iwasa, 1991). According to the analyses based on stochastic dynamic programming, there is a curve indicating a “critical size” exists. Before this curve, the plant should continue producing leaves irrespective of the current conditions (Figure 6). However, after the critical size is reached, the optimal behavior should differ depending on the current light conditions: the plant should grow under good light conditions but stop growing and start reproductive activities under dark setting. Another curve exists for the second switching, after which the plant should produce no leaves, irrespective of the current environment (Figure 6).

Ken‐ichi Hironaka and colleagues applied a similar approach to *Drosophila* larvae (Hironaka et al., 2019; Hironaka & Morishita, 2017). Similar to the vegetative growth of plants, larvae eat and grow rapidly. They interpreted the growth response to fluctuating food supply conditions as an optimal schedule (Yamada et al., 2020). Before reaching the critical weight, the larvae grow in both environments. When larvae reach the critical weight, the growth behavior of *Drosophila* larvae becomes dependent on the current food level. Under conditions of high food availability, they continue to grow as quickly as possible. However, under low food availability (i.e., starvation conditions), larvae immediately reduce energy expenditure and start conserving resources already invested in material to be used for pupation, metamorphosis, and the adult body.

### 5.2 Robust development: Spatial arrangement of the sources of chemical signals

Development takes place under various perturbations, or environmental noise. Developmental mechanisms must be able to produce normal forms even under unpredictable noise. A question arises whether the developmental process observed is designed to be “robust” in the presence of noise. Yoshihiro Morishita and colleagues investigated this question by studying chick limb bud formation. The source of FGF signals exists at the distal tip of the limb bud, and SHH signals exist at a posterior location away from the distal tip. These two chemical signals form gradients that provide positional information to cells. Each cell in the tissue may be able to determine its location based on the concentrations it experiences and execute the next step as directed, followed by the proper morphogenesis of the entire tissue. However, because the processes include noises producing higher or lower concentrations of chemical signals, each cell receives “incorrect” information on its own location, resulting in blurred and shifted morphogenesis. Placing signal sources in an appropriate configuration enables the system to be more robust to random noise than in other configurations (Morishita & Iwasa, 2008b, 2011). Because developmental processes that are robust against noise are advantageous, the spatial arrangement of the two signal chemicals adopted in the chick limb bud may be close to the one that achieves the most robust development. In a series of papers, Morishita developed this idea and proposed information encoding/decoding concepts in developmental biology (Hironaka & Morishita, 2012; Morishita & Iwasa, 2011). Relating the effect of noise on biological processes to the design of the process is an important future research direction in developmental biology.

## 6 CONCLUDING REMARKS

In this review, we explained four examples of the mathematical models that have been developed over the last decade. However, even if we combine these new models with the classical well‐established models mentioned previously, we have not discovered all the mathematical concepts and techniques that help us understand developmental mechanisms. Novel models that are useful for understanding key developmental processes must be generated and tailored to particular questions by experimental developmental biologists, possibly in collaboration with theoreticians. We encourage developmental biologists to collaborate with theoreticians from diverse backgrounds, such as mathematics, physics, engineering, informatics, and mathematical biology.
